# The Role of C-Reactive Protein and Fibrinogen in the Development of Intracerebral Hemorrhage: A Mendelian Randomization Study in European Population

**DOI:** 10.3389/fgene.2021.608714

**Published:** 2021-02-04

**Authors:** Biyan Wang, Xiaoyu Zhang, Di Liu, Jie Zhang, Mingyang Cao, Xin Tian, Isinta Elijah Maranga, Xiaoni Meng, Qiuyue Tian, Feifei Tian, Weijie Cao, Wei Wang, Manshu Song, Youxin Wang

**Affiliations:** ^1^Beijing Key Laboratory of Clinical Epidemiology, School of Public Health, Capital Medical University, Beijing, China; ^2^School of Medical and Health Sciences, Edith Cowan University, Joondalup, WA, Australia

**Keywords:** C-reactive protein, fibrinogen, single-nucleotide polymorphisms, Mendelian randomization, intracerebral hemorrhage

## Abstract

**Background:** The causal association of C-reactive protein (CRP) and fibrinogen on intracerebral hemorrhage (ICH) remains uncertain. We investigated the causal associations of CRP and fibrinogen with ICH using two-sample Mendelian randomization.

**Method:** We used single-nucleotide polymorphisms associated with CRP and fibrinogen as instrumental variables. The summary data on ICH were obtained from the International Stroke Genetics Consortium (1,545 cases and 1,481 controls). Two-sample Mendelian randomization estimates were performed to assess with inverse-variance weighted and sensitive analyses methods including the weighted median, the penalized weighted median, pleiotropy residual sum and outlier (MR-PRESSO) approaches. MR-Egger regression was used to explore the pleiotropy.

**Results:** The MR analyses indicated that genetically predicted CRP concentration was not associated with ICH, with an odds ratio (OR) of 1.263 (95% CI = 0.935–1.704, *p* = 0.127). Besides, genetically predicted fibrinogen concentration was not associated with an increased risk of ICH, with an OR of 0.879 (95% CI = 0.060–18.281; *p* = 0.933). No evidence of pleiotropic bias was detected by MR-Egger. The findings were overall robust in sensitivity analyses.

**Conclusions:** Our findings did not support that CRP and fibrinogen are causally associated with the risk of ICH.

## Introduction

Globally, stroke is a leading cause of death with a high societal burden in most regions (GBD 2015 Mortality and Causes of Death Collaborators, 2016). Among adults, the risk of stroke from the age of 25 years is approximately 25% (Feigin et al., 2018). Hemorrhagic stroke (HS) as a subtype of stroke carries high morbidity and mortality rates (Stokum et al., 2015), and intracerebral hemorrhage (ICH) is by far the most common type of HS (Qureshi et al., 2009). Inflammation plays an important part in pathogenesis of stroke by influencing the development of atherosclerosis and plaque instability (Barone and Feuerstein, 1999; Scirica and Morrow, 2006).

C-reactive protein (CRP) and fibrinogen, considered as well-proven clinical markers of systemic inflammation, are acute-phase protein synthesized by hepatocytes against inflammation (Dalmon et al., 1993; Pepys and Hirschfield, 2003) and can increase the risk of cardiovascular disease (Scirica and Morrow, 2006; Zhang et al., 2014) and stroke (Coull et al., 1991; Cao et al., 2007; Jiménez et al., 2015). Significantly increased levels of fibrinogen are commonly found in patients with stroke, suggesting that fibrinogen is elevated before thrombotic incidents occur and is a risk factor for stroke (Coull et al., 1991). However, Roudbary et al. (2011) revealed that CRP concentration was not improved in patients with HS. No associations of CRP and fibrinogen with ICH were identified in a nested case-control study (Karim et al., 2020). The observational epidemiologic studies on the associations of CRP and fibrinogen with ICH showed inconsistent results (Coull et al., 1991; Roudbary et al., 2011). Furthermore, potential unmeasured confounders and reverse causation bias in observational studies limit the ability to ascertain causal inferences.

Mendelian randomization (MR) is a genetic epidemiological method to explore the association between the exposure and outcome, using genetic variants as instrumental variables (IVs) for the exposure (Smith and Ebrahim, 2003). Because of the independent segregation and randomized assignment of alleles at meiosis, MR approach can control potential confounders and reverse causation, making stronger causal inference (Lawlor et al., 2008). Therefore, we conducted two-sample MR analysis to assess the causal relationships of CRP and fibrinogen in the development of ICH in European population.

## Materials and Methods

### Study Design and Data Sources

A two-sample MR approach was used to investigate the causal effects of CRP and fibrinogen on the risk of ICH. The study design is under the assumption that the genetic variants are associated with CRP and fibrinogen, but not with confounders. Besides, the genetic variants affect risk of ICH only through exposure and not through any alternative pathways.

Information on genetic variants associated with level of CRP was collected from a meta-analysis of genome-wide association study (GWAS), which is currently the largest study attempted to identify genetic variants in relation to CRP concentration involving 204,402 individuals from 88 previous population-based cohort studies (Ligthart et al., 2018). In genetic variants associated with fibrinogen, we used previously published genetic variants of a GWAS meta-analysis involving more than 100,000 subjects (Sabater-Lleal et al., 2013).

Summary statistics data on associations of genetic variants with ICH were obtained from the published GWAS meta-analysis by the International Stroke Genetics Consortium (ISGC) of 3,026 participants (1,545 cases and 1,481 controls; Woo et al., 2014). All data in our MR analyses were restricted to individuals of European ancestry only.

### Genetic Variants

We used single-nucleotide polymorphisms (SNPs) published previously, which reached genome-wide significance (*p* < 5 × 10^−8^) for CRP and fibrinogen concentrations as MR IVs. The selected SNPs were independent, namely, not in linkage disequilibrium (*r*^2^ < 0.2). Nineteen SNPs (11 for CRP and 8 for fibrinogen) were not presented in ISGC datasets. For the unavailable SNPs in outcome datasets, we replaced them with proxy SNPs. The proxy SNPs in linkage disequilibrium (*r*^2^ > 0.8) were identified for two SNPs. Accordingly, 42 SNPs for CRP and 16 SNPs for fibrinogen were included in the analysis of ICH. The summary genetic association data are reported in Supplementary Table S1.

### Mendelian Randomization Analysis

We performed two-sample MR analyses to estimate the associations of CRP and fibrinogen with ICH using summarized data. Causal effects on ICH of CRP and fibrinogen concentrations were estimated using the conventional inverse-variance weighted (IVW) method (Burgess et al., 2013). We also conducted sensitivity analyses using the weighted median (WM), the penalized weighted median (PWM), and pleiotropy residual sum and outlier (MR-PRESSO) methods (Bowden et al., 2015, 2016; Verbanck et al., 2018). For MR-Egger regression analysis, we assessed directional pleiotropy based on its intercepts (Burgess and Thompson, 2017). A leave-one-out analysis (omitted one SNP in turn) was performed to test the influence of outlying values (Burgess and Thompson, 2017). Heterogeneity of individual genetic variants was evaluated by Cochran’s *Q* test. All results are presented as an odds ratio (OR) with 95% confidence interval (CI) of the outcomes per predicted increase in CRP and fibrinogen concentrations. The associations of each SNP with CRP and fibrinogen concentrations are further plotted compared to their effects for the outcomes. All analyses were performed by the TwoSampleMR and MR-PRESSO packages with R version 4.0.2.

## Results

### Causal Association of CRP With ICH

The results of associations between genetically determined CRP and fibrinogen and the risk of ICH were presented in Table 1. Genetic predisposition to CRP levels were not observed to be statistically significantly associated with ICH by performing IVW method (OR = 1.263, 95% CI = 0.935–1.704, *p* = 0.127). The lack of causal association remained in all sensitivity analyses (all *p* > 0.05; Table 1).

**Table 1:** Mendelian randomization (MR) estimates of exposure with intracerebral hemorrhage from the inverse-variance weighted (IVW) and sensitivity analysis.

Phenotype	IVs (SNPs)	OR (95% CI)	*p*
**CRP**			
IVW	42	1.263 (0.935–1.704)	0.127
Weighted median	42	1.458 (0.977–2.175)	0.065
Penalized weighted median	42	1.466 (0.957–2.247)	0.079
MR_Egger	42	1.432 (0.906–2.266)	0.133
MR-PRESSO	42	1.236 (0.950–1.522)	0.154
**Fibrinogen**			
IVW	16	0.879 (0.042–18.281)	0.933
Weighted median	16	0.438 (0.016–11.771)	0.623
Penalized weighted median	16	0.438 (0.014–13.561)	0.637
MR_Egger	16	1.663 (0.004–746.651)	0.872
MR-PRESSO	16	1.221 (−1.893 to 4.335)	0.901

CRP, C-reactive protein; IVW, inverse-variance weighted; MR, Mendelian randomization; WM, weighted median; PWM, penalized weighted median; OR, odds ratio; MR-PRESSO, pleiotropy residual sum and outlier; CI, confidence interval.

**Figure 1:**
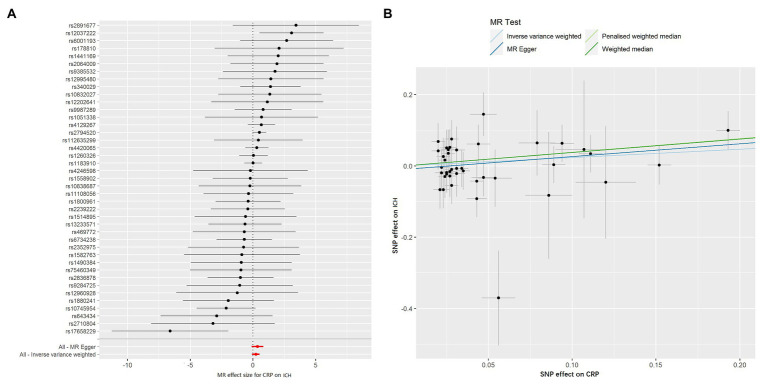
Forest plots and scatter plots of CRP-associated SNPs potential effects on intracerebral hemorrhage (ICH). Forest plot **(A)** shows the odds ratio (OR) with a horizontal line representing 95% CI for the CRP-associated SNP allele for ICH risk. Scatter plot **(B)** shows the per-allele association with ICH risk plotted against the per-allele association with 1 SD of CRP (vertical and horizontal black lines presenting the 95% CI of OR for each SNP), with the slope of each line corresponding to estimated Mendelian randomization (MR) effect per method.

The MR-Egger method showed no evidence of directional pleiotropy for the association of CRP with ICH [odds (intercept), −0.010; *p* = 0.480; Table 2]. For IVs, MR-PRESSO did not detect any potential outliers. Likewise, no heterogeneity was observed among individual SNPs of CRP for ICH (*Q* = 36.775, *p* = 0.616, Table 2). We calculated the individual and pooled MR estimates between each CRP-related SNP and the risk for ICH shown as forest plots and scatter plots in Figure 1. The result of leave-one-out sensitivity analysis showed that the association between CRP and ICH was not substantially driven by any individual SNP (Supplementary Figure S1).

**Table 2:** Heterogeneity tests and MR-Egger intercept of CRP and fibrinogen causally linked to ICH.

Outcome	Exposure	Intercept	*p*^a^	Cochran’s *Q*	Q_df	*p*^b^
ICH	CRP	−0.010	0.480	36.775	40	0.616
ICH	Fibrinogen	−0.008	0.815	28.028	15	0.045

a Value of *p* for MR-Egger intercept.

b Value of *p* for heterogeneity tests by performing inverse-variance weighted method. CRP, C-reactive protein; ICH, intracerebral hemorrhage.

### Causal Association of Fibrinogen With ICH

Regarding fibrinogen, we found no causal effect of genetically instrumented fibrinogen on ICH (OR = 0.879, 95% CI = 0.042–18.281, *p* = 0.933). No significant association was observed for ICH in sensitivity analyses that were performed by WM, PWM, MR-PRESSO, and MR-Egger methods (Table 1).

**Figure 2:**
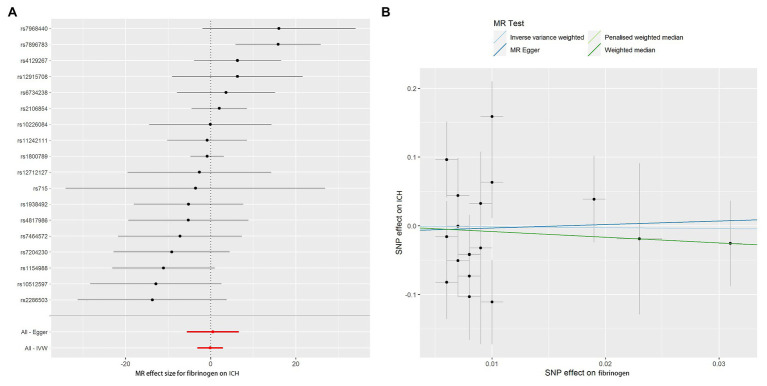
Forest plots and scatter plots of fibrinogen-associated SNPs potential effects on ICH. Forest plot **(A)** shows the odds ratio (OR) with a horizontal line representing 95% CI for the fibrinogen-associated SNP allele for ICH risk. Scatter plot **(B)** shows the per-allele association with ICH risk plotted against the per-allele association with 1 SD of fibrinogen (vertical and horizontal black lines presenting the 95% CI of OR for each SNP), with the slope of each line corresponding to estimated Mendelian randomization (MR) effect per method.

The MR-Egger method showed no evidence of directional pleiotropy for the association of fibrinogen with ICH (Table 2). For IVs, MR-PRESSO did not detect any potential outliers. We calculated the individual and pooled MR estimates between each fibrinogen-related SNP and risk for ICH shown as forest plots and scatter plots in Figure 2. Furthermore, analysis on leaving out each SNP revealed that the inverse association between fibrinogen concentrations and ICH was not substantially driven by any individual SNP (Supplementary Figure S2). However, the Cochran *Q* statistic was 28.028 with an associated *p* < 0.05, suggesting some heterogeneity in the effect estimates of fibrinogen and ICH (Table 2), but there was no clear evidence of directional pleiotropy (*p* for intercept > 0.05, Table 2).

## Discussion

In the present study, we assessed whether high circulating levels of CRP and fibrinogen are causally associated with ICH using two-sample MR analysis in European population. In the present study using publicly available summary statistics data, we did not find CRP and fibrinogen levels might increase ICH risk. The findings were overall robust in sensitivity analyses.

Apart from being markers of systemic inflammation, CRP and fibrinogen are acute-phase protein induced by proinflammatory cytokine contributing to host defense against infection (Dalmon et al., 1993; Pepys and Hirschfield, 2003). Previous studies investigated associations between CRP and fibrinogen and ICH but reported inconsistent results. A large-scale cohort study found that CRP and fibrinogen were not associated with a significantly greater risk of HS (Jiménez et al., 2016), while a retrospective cohort study suggested that increased CRP was a significant risk factor for in-hospital mortality among patients with cardiovascular disease including ICH (Yoshinaga et al., 2017).

In our analysis, we did not observe the relationships of CRP and fibrinogen with ICH. These findings suggested that the role of CRP and fibrinogen may be less important in causing the risk of ICH. A previous MR study indicated that CRP concentration itself was unlikely to be even a modest causal factor in coronary heart disease (Wensley et al., 2011). Our findings corroborate earlier studies that showed CRP had no clear effect on ICH risk (Liu et al., 2014). Similar results were also found in a meta-analysis consisting of six population-based prospective studies (Georgakis et al., 2019). Another meta-analysis has also suggested that elevated baseline CRP levels exhibited no clear effect on HS (Zhou et al., 2016). However, evidence from a few prospective studies showed that CRP level in HS patients was significantly elevated (Das et al., 2014; Xue et al., 2017).

Fibrinogen participates in platelet aggregation, thrombogenic activity, atherogenesis, and inflammation, and the role of fibrinogen is probably various in the different subtypes of stroke. Our findings were supported by previous studies, which also reported that elevated levels of fibrinogen did not exhibit suggestive evidence of association with HS (Alvarez-Perez et al., 2011). In line with our results, no significant association between fibrinogen and ICH was observed in observational studies (Woodward et al., 2005; Welsh et al., 2008; Folsom et al., 2016). However, greater plasma fibrinogen concentration was associated with increased risk of ICH in these prospective studies (Sato et al., 2006; Sturgeon et al., 2008). These findings should be interpreted cautiously as higher CRP and fibrinogen levels may reflect subclinical infection, chronic infectious diseases, preexisting disease, and socioeconomic or lifestyle characteristics. Besides, these opposite results may be due to different study populations and ethnic groups (Iso et al., 2012; Shi et al., 2016).

The pathogenesis of the associations of CRP and fibrinogen with the risk of ICH is unclear. CRP plays a direct role in the pathogenesis of atherosclerosis and is upregulated significantly in atheromatous plaques, where it may promote low-density lipoprotein cholesterol uptake by macrophages (Torzewski et al., 2000). Moreover, these inconsistent previous results may be due to reverse causal bias or confounders from atherosclerosis (Libby et al., 2011) or inflammation (Hartwig et al., 2017). One possible explanation is that the previous finding was a false-positive outcome because the effect of confounding was not controlled for, whereas in our studies, the genetic variants associated with exposure explained a larger proportion of variance, showing the true relationship of CRP and fibrinogen with ICH. Another possible explanation is that a mass of variants resulted in greater pleiotropy potential, which may have diluted the association in our analysis.

The major strengths of this study are using data from large-scale GWAS studies and ISGC collaboration. We used a two-sample MR approach assessing CRP and fibrinogen levels in relation to the risk of ICH in European-descent individuals, which reduces bias of population stratification. Moreover, in terms of the MR analysis, we performed conventional IVW, WM, PWM, MR-PRESSO, and MR-Egger methods to avoid reverse causation and to reduce other confounding factors. Lastly, there is no strong evidence of pleiotropic effects for the genetic instruments, suggesting there was less likelihood of CRP and fibrinogen-related SNPs are associated with other phenotypes.

The present study also has some limitations. Interpreting the magnitude of estimates for the effect of CRP and fibrinogen on ICH risk requires caution. First, stratified analyses or analyses adjusted for other covariates were not possible on the account of using the available summary statistics datasets. In addition, the genetic IVs accounted for approximately 7.0% of the total variation in CRP and 3.7% of plasma fibrinogen variation (Sabater-Lleal et al., 2013; Ligthart et al., 2018), which might be low for the use as IVs, and any bias from weak instruments was in the direction of the null (Pierce and Burgess, 2013). Nevertheless, MR analysis likely reflects lifelong exposure to elevated CRP and fibrinogen levels. However, it is possible that only exposure in a specific window of time (e.g., early life) affects ICH risk. Lastly, we used a relatively small sample size to explore the causal relationship between CRP, fibrinogen, and ICH with the power of less than 0.90. Thus, the nonsignificant but still suggestive associations between CRP and fibrinogen levels and ICH risk should be further validated in future studies with larger independent populations and larger datasets offering greater statistical power.

In conclusion, these MR analyses did not find evidence to support the causal relationship between CRP and fibrinogen with ICH. The results add to the burgeoning evidence that refutes the harmful role of CRP and fibrinogen in ICH. Further research is required to clarify this finding, using larger samples for undertaking “adjusted” MR analyses.

## Data Availability Statement

The original contributions presented in the study are included in the article/Supplementary Material, further inquiries can be directed to the corresponding authors.

## Author Contributions

BW and XZ drafted the manuscript. YW, MS, DL, JZ, MC, XT, IM, XM, QT, FT, WC, and WW critically revised the manuscript. All authors contributed to the article and approved the submitted version.

### Conflict of Interest

The authors declare that the research was conducted in the absence of any commercial or financial relationships that could be construed as a potential conflict of interest.
