# Supplementary material for: The Role of C-Reactive Protein and Fibrinogen in the Development of Intracerebral Hemorrhage: A Mendelian Randomization Study in European Population
Source: Front Genet. 2021 Feb 4;12:608714. doi: 10.3389/fgene.2021.608714 (PMC7890085; doi:10.3389/fgene.2021.608714)
Supplement: Supplementary file 1 [file Table_1.doc]

**Table 1.** Summary statistics for the genetic variants associated with the CRP and fibrinogen investigated for an association with intracerebral hemorrhage in the present Mendelian randomization study

|  |  |  |  |  | Exposure | |  | Outcome results | |  |
| --- | --- | --- | --- | --- | --- | --- | --- | --- | --- | --- |
| Exposure | SNP | Chr | Nearest gene | EA | *β* | SE | *P* value | *β* | SE | *P* value |
| CRP | rs2794520 | 1 | *CRP* | C | 0.193 | 0.007 | 9.5×10^-189^ | 0.099 | 0.053 | 0.064 |
| CRP | rs1183910 | 12 | *HNF1A* | G | 0.152 | 0.007 | 3.3×10^-113^ | 0.002 | 0.054 | 0.965 |
| CRP | rs4420065 | 1 | *LEPR* | C | 0.111 | 0.007 | 3.2×10^-64^ | 0.034 | 0.052 | 0.515 |
| CRP | rs4129267 | 1 | *IL6R* | C | 0.094 | 0.007 | 1.1×10^-47^ | 0.063 | 0.052 | 0.226 |
| CRP | rs1260326 | 2 | *GCKR* | T | 0.089 | 0.007 | 5.4×10^-43^ | -0.003 | 0.051 | 0.946 |
| CRP | rs6734238 | 2 | *IL1F10* | G | 0.047 | 0.007 | 3.4×10^-13^ | -0.032 | 0.052 | 0.538 |
| CRP | rs9987289 | 8 | *PPP1R3B** | G | 0.079 | 0.011 | 2.3×10^-12^ | -0.064 | 0.092 | 0.484 |
| CRP | rs10745954 | 12 | *ASCL1** | A | 0.043 | 0.006 | 1.6×10^-11^ | 0.092 | 0.051 | 0.074 |
| CRP | rs1800961 | 20 | *HNF4A* | C | 0.120 | 0.018 | 2.3×10^-11^ | -0.046 | 0.158 | 0.771 |
| CRP | rs340029 | 15 | *RORA* | T | 0.044 | 0.007 | 2.6×10^-11^ | -0.061 | 0.054 | 0.257 |
| CRP | rs12037222 | 1 | *PABPC4* | A | 0.047 | 0.008 | 4.5×10^-10^ | -0.144 | 0.061 | 0.017 |
| CRP | rs13233571 | 7 | *BCL7B* | A | 0.054 | 0.010 | 2.8×10^-8^ | 0.034 | 0.080 | 0.667 |
| CRP | rs2836878 | 21 | *DSCR2** | G | 0.040 | 0.007 | 4.0×10^-8^ | 0.042 | 0.057 | 0.458 |
| CRP | rs4903031 | 14 | *RGS6* | A | -0.046 | 0.008 | 4.6×10^-8^ | 0.0215 | 0.0649 | 0.7406 |
| CRP | rs469772 | 1 | *ZNF644* | T | -0.031 | 0.005 | 5.54×10^-12^ | -0.0443 | 0.0664 | 0.5045 |
| CRP | rs12995480 | 2 | *TMEM18* | T | -0.031 | 0.005 | 1.24×10^-10^ | -0.0043 | 0.0513 | 0.934 |
| CRP | rs4246598 | 2 | *FABP1* | A | 0.022 | 0.004 | 5.11×10^-10^ | 0.0285 | 0.0584 | 0.626 |
| CRP | rs9284725 | 2 | *IL1R1* | C | 0.027 | 0.004 | 7.34×10^-11^ | 0.0503 | 0.0515 | 0.3288 |
| CRP | rs1441169 | 2 | *IKZF2* | G | -0.025 | 0.004 | 2.27×10^-11^ | 0.0188 | 0.0564 | 0.7387 |
| CRP | rs2352975 | 3 | *TRAIP* | C | 0.025 | 0.004 | 6.43×10^-10^ | 0.3704 | 0.1326 | 0.005224 |
| CRP | rs9271608 | 6 | *HLA-DQA1* | G | 0.042 | 0.005 | 2.33×10^-17^ | -0.0263 | 0.0524 | 0.6158 |
| CRP | rs12202641 | 6 | *FRK* | T | -0.023 | 0.004 | 3.00×10^-10^ | 0.0232 | 0.0513 | 0.651 |
| CRP | rs1490384 | 6 | *C6orf173* | T | -0.025 | 0.004 | 2.65×10^-12^ | -0.0457 | 0.0549 | 0.405 |
| CRP | rs9385532 | 6 | *L3MBTL3* | T | -0.026 | 0.004 | 1.90×10^-11^ | -0.0549 | 0.0517 | 0.2882 |
| CRP | rs1880241 | 7 | *IL6* | G | -0.028 | 0.004 | 8.41×10^-14^ | 0.067 | 0.0528 | 0.2048 |
| CRP | rs2710804 | 7 | *KIAA1706* | C | 0.021 | 0.004 | 1.30×10^-8^ | 0.0519 | 0.0512 | 0.3112 |
| CRP | rs2064009 | 8 | *TRPS1* | C | -0.027 | 0.004 | 2.28×10^-14^ | 0.0686 | 0.0513 | 0.1816 |
| CRP | rs2891677 | 8 | *NSMCE2* | C | -0.02 | 0.004 | 1.59×10^-8^ | -0.0666 | 0.0524 | 0.2032 |
| CRP | rs643434 | 9 | *ABO* | A | 0.023 | 0.004 | 1.02×10^-9^ | -0.0164 | 0.0552 | 0.7663 |
| CRP | rs1051338 | 10 | *LIPA* | G | 0.024 | 0.004 | 2.27×10^-9^ | 0.0349 | 0.0547 | 0.5229 |
| CRP | rs10832027 | 11 | *ARNTL* | G | -0.026 | 0.004 | 4.43×10^-12^ | -0.0073 | 0.0646 | 0.9105 |
| CRP | rs10838687 | 11 | *MADD* | G | -0.031 | 0.004 | 9.12×10^-13^ | 0.0194 | 0.0519 | 0.7078 |
| CRP | rs7121935 | 11 | *STARD10* | A | -0.022 | 0.004 | 5.28×10^-9^ | -0.0101 | 0.0511 | 0.8434 |
| CRP | rs11108056 | 11 | *METAP2* | G | -0.028 | 0.004 | 5.42×10^-14^ | 0.0143 | 0.0527 | 0.7868 |
| CRP | rs4774590 | 15 | *DMXL2* | A | -0.022 | 0.004 | 2.71×10^-8^ | -0.0072 | 0.052 | 0.8903 |
| CRP | rs1558902 | 16 | *FTO* | A | 0.034 | 0.004 | 5.20×10^-20^ | 0.0418 | 0.0524 | 0.425 |
| CRP | rs4092465 | 18 | *ONECUT2* | A | -0.027 | 0.004 | 3.11×10^-10^ | 0.0302 | 0.0595 | 0.6116 |
| CRP | rs2315008 | 20 | *ZGPAT* | T | -0.023 | 0.004 | 5.36×10^-10^ | 0.0427 | 0.0577 | 0.4585 |
| CRP | rs2836878 | 21 | *DSCR2* | G | 0.043 | 0.004 | 7.71×10^-26^ | 0.0752 | 0.053 | 0.1555 |
| CRP | rs6001193 | 22 | *TOMM22* | G | -0.028 | 0.004 | 6.53×10^-14^ | -0.0826 | 0.1779 | 0.6425 |
| CRP | rs75460349 | 1 | *ZDHHC18* | A | 0.086 | 0.014 | 4.50×10^-10^ | 0.016 | 0.0561 | 0.7753 |
| CRP | rs1514895 | 3 | *EIF5A2* | A | -0.027 | 0.004 | 2.70×10^-9^ | -0.0462 | 0.193 | 0.8107 |
| CRP | rs1189402 | 15 | *ONECUT1* | A | 0.025 | 0.004 | 3.90×10^-9^ | 0.0215 | 0.0649 | 0.7406 |
| Fibrinogen | rs1938492 | 1 | *LEPR* | A | 0.008 | 0.001 | 5.3×10^-14^ | -0.0416 | 0.0526 | 0.4296 |
| Fibrinogen | rs4129267 | 1 | *IL6R* | T | 0.010 | 0.001 | 1.2×10^-19^ | 0.0632 | 0.0522 | 0.2264 |
| Fibrinogen | rs12712127 | 2 | *IL1R1*/IL1R2* | A | 0.006 | 0.001 | 2.7×10^-08^ | -0.0160 | 0.0517 | 0.7566 |
| Fibrinogen | rs6734238 | 2 | *IL1F10*/IL1RN* | A | -0.009 | 0.001 | 5.8×10^-19^ | -0.0326 | 0.0529 | 0.5385 |
| Fibrinogen | rs715 | 2 | *CPS1* | T | 0.009 | 0.001 | 2.0×10^-11^ | -0.0322 | 0.1399 | 0.8179 |
| Fibrinogen | rs1154988 | 3 | *MSL2*/PCCB* | A | -0.010 | 0.001 | 9.6×10^-17^ | 0.1109 | 0.0614 | 0.07095 |
| Fibrinogen | rs1800789 | 4 | *FGB* | A | 0.031 | 0.001 | 1.7×10^-127^ | -0.0257 | 0.0622 | 0.6801 |
| Fibrinogen | rs11242111 | 5 | *131783957* | A | 0.023 | 0.002 | 1.6×10^-21^ | -0.0189 | 0.1101 | 0.8638 |
| Fibrinogen | rs2106854 | 5 | *C5orf56/IRF1* | T | -0.019 | 0.001 | 1.7×10^-48^ | -0.0386 | 0.0630 | 0.5399 |
| Fibrinogen | rs10226084 | 7 | *SN×13*/PRPS1L1* | T | -0.007 | 0.001 | 5.1×10^-10^ | 0.0005 | 0.0514 | 0.9916 |
| Fibrinogen | rs2286503 | 7 | *TOMM7* | T | -0.006 | 0.001 | 6.9×10^-09^ | 0.0823 | 0.0536 | 0.1248 |
| Fibrinogen | rs7464572 | 8 | *PLEC1* | C | -0.007 | 0.001 | 1.3×10^-09^ | 0.0506 | 0.0520 | 0.3302 |
| Fibrinogen | rs7896783 | 10 | *JMJD1C* | A | -0.010 | 0.001 | 8.9×10^-22^ | -0.1589 | 0.0512 | 0.001914 |
| Fibrinogen | rs7968440 | 12 | *DIP2B* | A | 0.006 | 0.001 | 2.7×10^-08^ | 0.0963 | 0.0551 | 0.08033 |
| Fibrinogen | rs12915708 | 15 | *SPPL2A* | C | -0.007 | 0.001 | 6.9×10^-10^ | -0.0440 | 0.0548 | 0.4224 |
| Fibrinogen | rs7204230 | 16 | *CHD9* | T | 0.008 | 0.001 | 1.2×10^-10^ | -0.0733 | 0.0556 | 0.1874 |
| Fibrinogen | rs10512597 | 17 | *CD300LF* | T | -0.008 | 0.001 | 9.9×10^-09^ | 0.1031 | 0.0630 | 0.1014 |
| Fibrinogen | rs4817986 | 21 | *PSMG1* | T | -0.008 | 0.001 | 2.5×10^-11^ | 0.0420 | 0.0577 | 0.466 |

CRP, C-reactive protein, Chr: chromosome; EA: effect allele; NEA: non-effect allele; SE: standard error; SNP: single-nucleotide polymorphism, *More than 60kb.
