# Supplementary figures and images for: The Role of C-Reactive Protein and Fibrinogen in the Development of Intracerebral Hemorrhage: A Mendelian Randomization Study in European Population
Source: Front Genet. 2021 Feb 4;12:608714. doi: 10.3389/fgene.2021.608714 (PMC7890085; doi:10.3389/fgene.2021.608714)

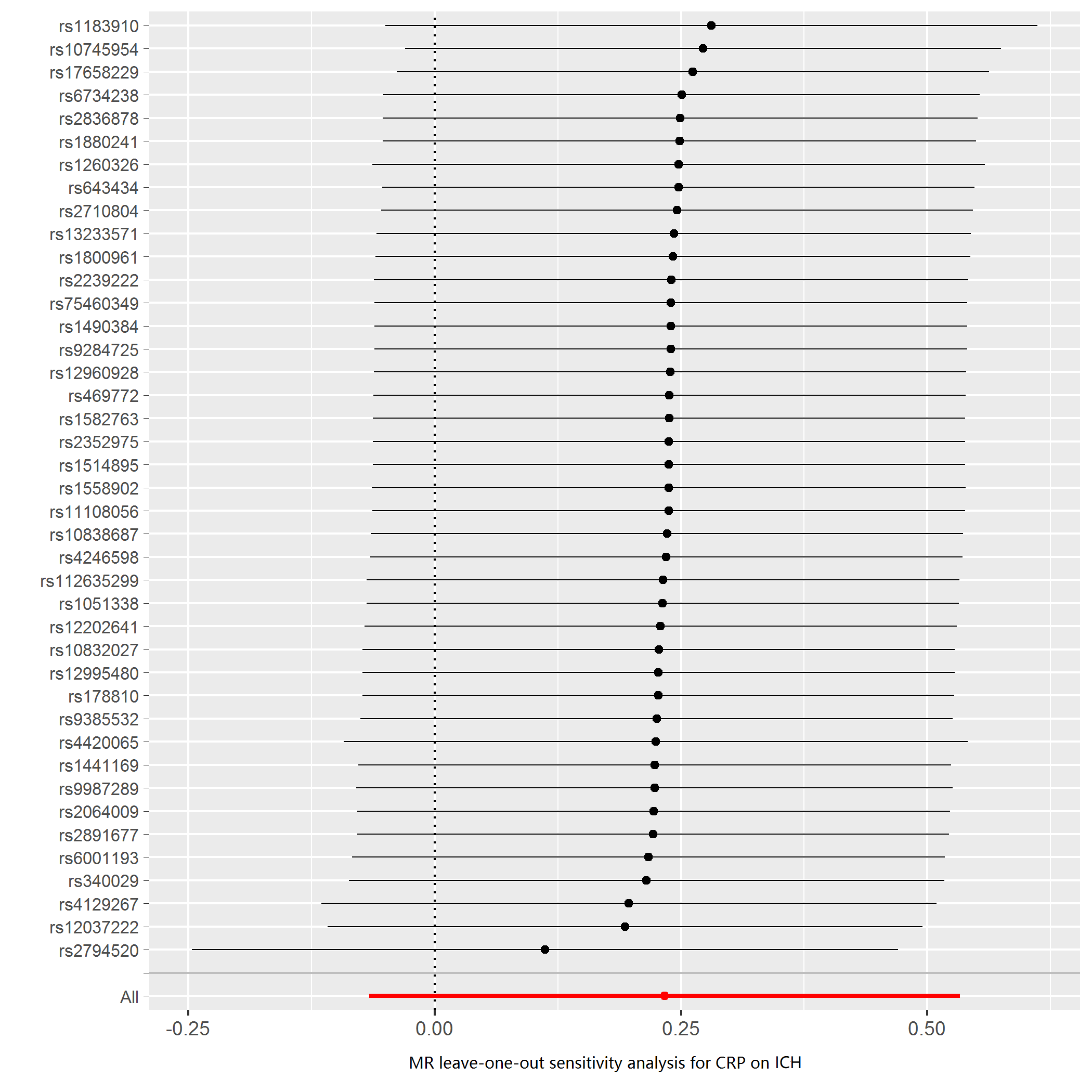

Supplement: Supplementary file 2 [file Image_1.tiff]

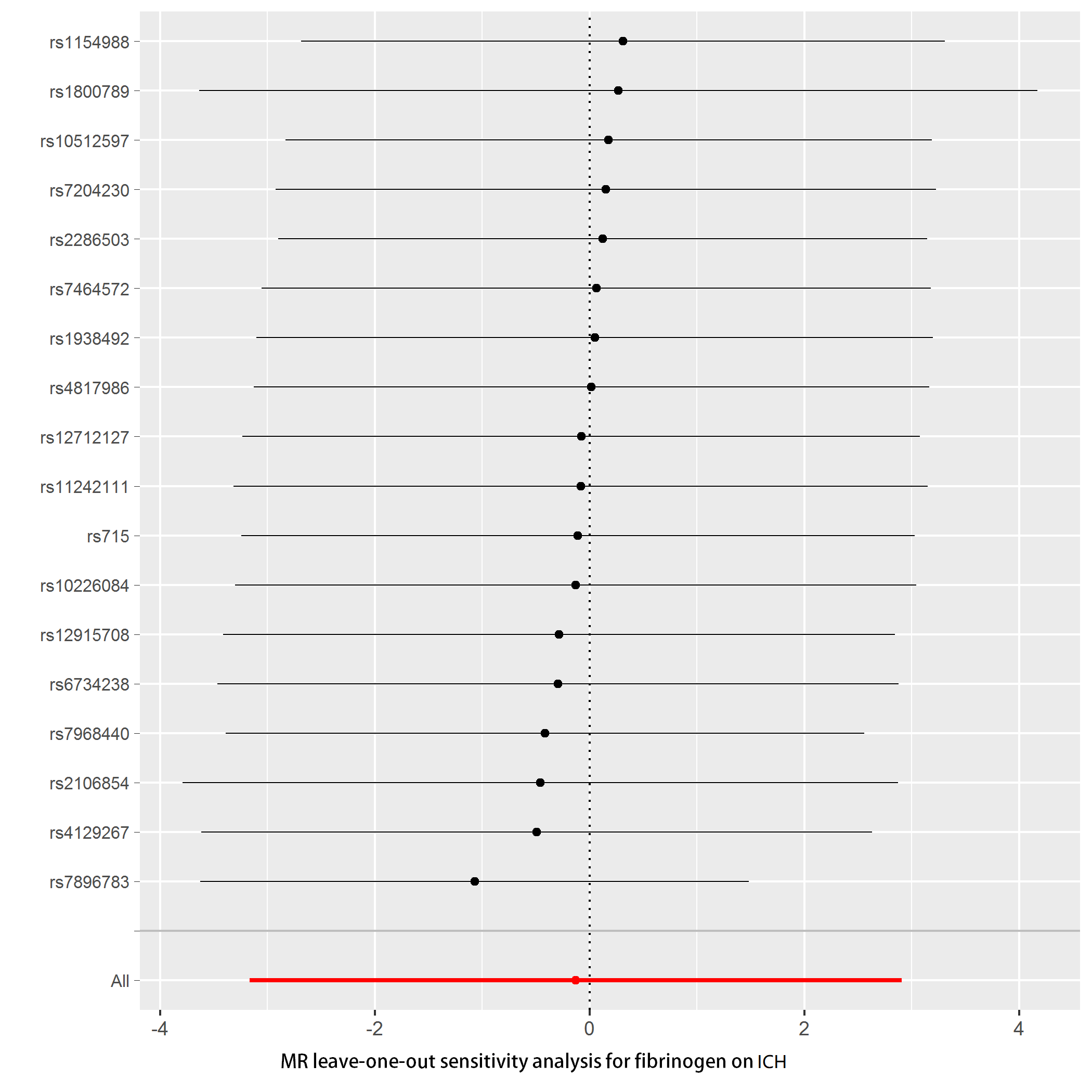

Supplement: Supplementary file 3 [file Image_2.tiff]
